# Comprehensive evaluation of *Balanites aegyptiaca* extracts: in vitro bioactivities and in vivo antimicrobial efficacy and hematological impact on broiler chickens challenged with *E. coli* O78 and *Clostridium perfringens* type A

**DOI:** 10.1007/s11259-026-11152-0

**Published:** 2026-04-07

**Authors:** Asmaa Ezzat, Heba M. Salem, Sara H. Mahmoud, Ahmed Mostafa, Mohamed A. Ali, Khaled Mahmoud, Nasser S. Flefil, Tarek N. Soliman, Asmaa Negm El-Dein

**Affiliations:** 1 Chemistry of Natural and Microbial Products Department, Pharmaceutical and Drug Industries Research Institute, National Research Centre, Dokki, Giza, Egypt; 2 Department of Poultry Diseases, Faculty of Veterinary Medicine, Cairo University, Giza, 12211 Egypt; 3 Department of Diseases of Birds, Rabbits, Fish & their Care & Wildlife, School of Veterinary Medicine, Badr University in Cairo (BUC), Badr City, Cairo 11829 Egypt; 4 Center of Scientific Excellence for Infuenza Viruses, National Research Centre, Dokki, Giza, 12622 Egypt; 5 Pharmacognosy Department, Pharmaceutical Industries Research Institute, National Research Centre, 33 El Bohouth st. (former El Tahrir st.), Dokki, P.O 12622, Cairo, Egypt; 6 National Institute of Oceanography and Fisheries, NIOF, Cairo, Egypt; 7 Dairy Department, Food Industries and Nutrition Research Institute, National Research Centre, Dokki, Giza, 12622 Egypt

**Keywords:** *Balanites aegyptiaca*, Antiviral, Antitumor, Antibacterial, Broiler chicks, Necrotic enteritis

## Abstract

*Balanites aegyptiaca* (L.) Delile, commonly known as desert date, is a multipurpose medicinal plant widely used in traditional African and Middle Eastern medicine. Various parts of the plant, particularly the seeds and fruit, are used to treat infections, gastrointestinal disturbances, parasitic diseases, and inflammation. Its rich composition of bioactive compounds suggests a potential role in managing microbial infections in veterinary contexts, including poultry farming. The current study aimed to evaluate the in vitro biological activities and for the first time the in vivo antimicrobial efficacy of fruit extract, seed oil and its nanoemulsion against *Escherichia coli* O78 and *Clostridium perfringens* type A in experimentally infected broiler chickens.

Fruit extract, seed oil, and nanoemulsions were evaluated for antioxidant, antiviral, antitumor, and antimicrobial activities using standard in vitro assays. The minimum inhibitory concentrations (MICs) of the fruit extract, seed oil, and nanoemulsion against both bacterial strains were 15 mg, 100 µL, and 250 µL, respectively. Two separate in vivo experiments were performed using broiler chicks (*n* = 5 groups per experiment): Group 1 (negative control, uninfected), Group 2 (positive control, infected with *E. coli* in experiment 1 or *C. perfringens* in experiment 2), Group 3 (infected and treated with date extract), Group 4 (infected and treated with seed oil nanoemulsion), and Group 5 (infected and treated with conventional antibiotic). Clinical signs, mortality, postmortem lesions, and bacterial colonization in intestinal tissues were assessed. Hematological parameters were also measured to evaluate safety.

The fruit extract exhibited the strongest biological activity among the tested formulations, demonstrating pronounced antimicrobial, antiviral, and antitumor effects, while seed oil exerted the highest antioxidant fraction. In in vivo studies, treatment with fruit extract significantly reduced mortality, mitigated clinical symptoms and pathological lesions, and suppressed intestinal colonization by both *E. coli* O78 and *C. perfringens* type A. The seed oil nanoemulsion also exhibited moderate protective effects. No adverse changes were observed in hematological parameters across treatment groups, indicating the extract’s safety at the administered doses.

*B. aegyptiaca* exhibits potent antimicrobial and antioxidant properties and offers promising protective effects against enteric infections in poultry. Its efficacy profile support its potential as a natural alternative to antibiotics in poultry health management. These findings provide scientific validation for the traditional use of *B. aegyptiaca* and warrant further investigation into its mechanisms of action and optimal application strategies in veterinary medicine.

## Introduction

The overuse of conventional antibiotics in poultry production has contributed significantly to the emergence of antimicrobial resistance (AMR), posing a serious threat to both animal and public health. This challenge has necessitated an urgent shift toward natural alternatives that can effectively control pathogenic infections while preserving bird performance and food safety. Among these alternatives, herbal phytogenics, such as garlic, oregano, and cinnamon, have demonstrated promising antimicrobial, immunomodulatory, and digestive-enhancing properties in poultry systems (Abd El-Hack et al. 2022a, b; Salem et al. 2023; Ahmed et al. 2025).

As the efficacy of traditional antibiotics declines, natural compounds including plant extracts, essential oils, and antimicrobial peptides have gained increasing attention for their broad-spectrum activity, multiple mechanisms of action, and lower propensity to induce resistance (El-Saadony et al. 2022; Soliman et al. 2024). These bioactives hold strong potential to combat resistant infections and contribute to sustainable poultry production (Abd El-Hack et al. 2022c).

*Balanites aegyptiaca* (Zygophyllaceae), commonly known as the “desert date,” is a hardy shrub native to the arid regions of Africa and South Asia. It has been widely used in traditional medicine for its broad pharmacological properties, including antibacterial, antifungal, anti-inflammatory, antioxidant, and antiparasitic effects. Phytochemical investigations have revealed that its fruit extracts are rich in bioactive compounds such as oleic and palmitic acids, β-sitosterol, flavonoid glycosides, and phenolics, which are known for their antimicrobial and health-promoting activities (Ibrahim et al. 2022).

The medicinal relevance of *B. aegyptiaca* is multifaceted. Its seed extracts, rich in steroidal saponins and flavonoids, exhibited significant antidiabetic effects. In vitro and in vivo experiments (including phytochemical profiling and diabetic-relevant cell lines and models) confirmed their ability to enhance insulin secretion and improve glucose metabolism (Bhardwaj et al. 2024).

Various parts of the plant, including leaves, bark, and fruit, exhibit antimicrobial effects against a range of bacterial and fungal pathogens and have been traditionally used to treat skin infections, wounds, and sexually transmitted infections (Ezemokwe et al. 2020). It has also been reported to possess antiparasitic and anthelmintic activity, with efficacy against *Schistosoma* and *Leishmania* species (Jaheed et al. 2019). Anti-inflammatory and analgesic effects have been observed in its leaf and bark extracts, traditionally employed for the relief of joint pain, fever, and swelling. Furthermore, *B. aegyptiaca* has shown hepatoprotective activity, attributed to its ability to mitigate liver damage caused by toxins, supporting its use in the management of jaundice and liver disorders. Its rich antioxidant profile helps combat oxidative stress, reducing cellular damage from free radicals (Abdullahi et al. 2020). Additionally, it has been used as a mild laxative and digestive aid, and for enhancing wound healing when applied topically (Annan and Dickson 2008). Notably, recent studies suggest that it can enhance poultry productivity and overall bird performance (Bashir et al. 2020; Abdullahi and Idachaba 2024).

In vitro studies have demonstrated that methanolic fruit extracts of *B. aegyptiaca* possess potent antibacterial activity, inhibiting human pathogens such as *Acinetobacter johnsonii*,* Serratia marcescens*, and *Agrobacterium tumefaciens* at minimum inhibitory concentrations (MICs) around 62.5 µg/mL (Ibrahim et al. 2022). Hydroethanolic and kernel-derived extracts have also shown inhibitory effects against a range of bacterial and fungal strains (Anani et al. 2015). Despite its demonstrated efficacy in vitro and in various animal models (e.g., goats, rats), research on its application in poultry, especially broilers, remains scarce. A recent study from Nigeria found that dietary inclusion of soaked *B. aegyptiaca* fruit meal up to 7.5%, in combination with acidifiers, did not negatively impact hematological or biochemical parameters in broilers, indicating its safety at practical feeding levels (Abdullahi and Idachaba 2024).

*Escherichia coli* and *Clostridium perfringens* represent major pathogens in the poultry industry, causing colibacillosis and necrotic enteritis, respectively. These diseases are associated with significant economic losses due to increased mortality, reduced growth performance, and elevated treatment costs (Negm El-Dein et al. 2024; Shakal et al. 2024a, b).

*E. coli* O78 was selected as it is one of the most prevalent avian pathogenic *E. coli* (APEC) serotypes causing colibacillosis in broilers, with high morbidity and economic losses reported globally. Similarly, *C. perfringens* type A is the principal toxinotype responsible for necrotic enteritis in poultry, a major enteric disease with significant impact on performance and welfare. Focusing on these clinically relevant and economically significant pathogens amid the ongoing antibiotic resistance crisis underscores the importance of evaluating the therapeutic potential of natural extracts against major bacterial challenges in broiler production systems (Van Immerseel et al., 2004; Cooper and Songer 2009; Kim et al. 2020; Jamali et al. 2024).

However, the impact of fruit extract and seed oil nanoemulsion of *B. aegyptiaca* on broilers experimentally challenged with *E. coli* O78 and *C. perfringens* type A has not been previously explored. Therefore, the present study aims to evaluate, for the first time, the in vivo antimicrobial efficacy and hematological responses associated with the administration of fruit extract and seed oil nanoemulsion of *B. aegyptiaca* in broiler chickens challenged with *E. coli* O78 and *C. perfringens* type (A) This work seeks to determine the potential of *(B) aegyptiaca* as a natural and safe therapeutic agent for enhancing poultry health and disease resistance under pathogenic stress.

## Materials and methods

### Materials

Sodium caseinate was prepared from the skim milk and pectin was obtained from Sisco Research Laboratories Pvt. Ltd. (Mumbai, India). *Balanites* fruits were sourced from a local market in Aswan, Egypt. The authenticity of the fruits was confirmed by Prof. Dr. M. Gebali, an expert in plant taxonomy and Egyptian flora at the National Research Centre, Giza, Egypt. A voucher specimen (No. 201) was deposited in the herbarium of the Pharmacognosy Department, Faculty of Pharmacy, Cairo University, Egypt.

### Extraction of date fruits

A total of 250 g of the epicarp and mesocarp of date fruits was immersed in 1 L of 80% ethyl acetate for extraction. This solvent system was selected because ethyl acetate is effective for extracting non-polar and moderately polar compounds, while the aqueous fraction improves solubility of more polar metabolites, thereby broadening the range of phytochemicals recovered. This mixed-solvent approach has been successfully applied in plant extraction studies, including the recovery of phenolic classes such as anthocyanins from grape pomace (Pintać et al. 2018).

The mixture was then centrifuged, and the resulting supernatant was carefully collected. Solvent removal was performed using a rotary evaporator (Heidolph 2000, Germany) operated under a vacuum pressure of 0.90 bar at 50 °C until complete evaporation of the solvent. The resulting viscous extract was subsequently freeze-dried using a lyophilizer (Labconco Corporation, Kansas City, United States) at − 52 °C for 48 h under a pressure of 0.1 mPa. The dried date extract (DE) was then stored at − 18 °C until further use.

### Extraction of date seed oil

A volume of 150 mL of n-hexane was measured and transferred into a round-bottom flask. A 100 g seed powder was placed into a filter paper thimble, which was then inserted into the central chamber of the Soxhlet extractor. The system was heated to 70 °C, causing the n-hexane to evaporate and rise through the vertical arm into the condenser. The condensed solvent dripped onto the sample in the thimble, dissolving the oil content and subsequently siphoning back into the flask. This continuous extraction cycle was maintained for approximately four hours to maximize oil yield (Ogala et al. 2018).

### Gas chromatography-mass spectrometry (GC-FID) of seed oil

Gas chromatography (GC) was employed to analyze the fatty acid composition of the seed oil by evaluating the fatty acid methyl esters (FAMEs) derived from the lipid fractions. The procedure was adapted and modified from the methodology described by Gebremeskal et al. (2024). An HP 6890 Plus gas chromatograph (Hewlett-Packard, USA), equipped with a Supelco™ SP-2380 capillary column (60 m × 0.25 mm i.d., 0.20 μm film thickness; Sigma-Aldrich, USA), was used for FAME separation. The column temperature program began at 140 °C (held for 5 min), ramped to 240 °C at 4 °C/min, and was maintained at 240 °C for an additional 10 min. Helium served as the carrier gas at a flow rate of 1.2 mL/min. The injector and flame ionization detector (FID) were both maintained at 250 °C. A split injection mode with a 100:20 split ratio was used, with 1 µL of the FAME sample dissolved in n-hexane injected into the system. FAMEs were identified by comparing their retention times to those of known standards from the Supelco™ 37-Component FAME Mix. Fatty acid content was determined by calculating the relative percentage of each peak area to the total chromatographic peak area. This GC-based approach enabled accurate profiling and quantification of the fatty acid composition in lipid fractions extracted from seed oil.

## Nano-immobilization of date seed oil

### Preparation of sodium caseinate from cow milk

Fresh full cow milk was obtained from the Farm of the Faculty of Agriculture, Cairo University (Cairo, Egypt), and centrifuged at 2000 × g for 30 min at 4 °C using a Centrifuge Elecrem (Helmut Rink GmbH, Amtzell, Germany) to separate the cream and obtain skim milk. Sodium caseinate was then prepared from the skim milk following the method of Shazly et al. (2017), with slight modifications. The skim milk was acidified to pH 4.6 by the gradual addition of 1 M hydrochloric acid under continuous stirring at 25 °C. After 20 min of curd formation, the coagulated casein was collected by filtration using Whatman No. 40 filter paper. The resulting curd was washed with distilled water, then dissolved in 1 M sodium hydroxide and adjusted to pH 7.0. This precipitation and washing process was repeated four times to ensure purity. The final precipitate was dissolved again in 1 M NaOH, adjusted to pH 7.0, and then heated at 80 °C for 30 min to inactivate plasmin. The solution was subsequently dialyzed against distilled water to remove residual salts and lyophilized to obtain purified sodium caseinate.

### Physicochemical characteristics of sodium caseinate

The crude protein, moisture, ash, and crude fat contents of the sodium caseinate were determined using the Kjeldahl method, oven drying at 105 °C, dry combustion, and Soxhlet extraction, respectively. Color measurements were performed using a colorimeter (WSC–2B, IOT Instruments Co., Ltd., Shanghai, China) to determine the L^*^, a^*^, and b^*^ values of the samples. Each measurement was conducted in triplicate. The total color difference (ΔE) of the protein sample compared to a white standard was calculated using the following equation:

$$\triangle E=\sqrt{\left(L^\ast-L\right)^2+\left(\alpha^\ast-\alpha\right)^2+\left(b^\ast-b\right)^2}$$

L^*^, a^*^, and b^*^ represent the color values of the white standard, while L, a, and b correspond to the values measured for the protein samples. The reference values for the white standard were: L^*^ = 47.5, a^*^ = 2.4, and b^*^ = 7.5.

### Preparation of seed oil nanoemulsion

Date oil nanoemulsions were prepared by homogenizing date oil, sodium caseinate (NaCas), and pectin using an Ultra-Turrax T25 homogenizer at 15,000–24,000 rpm for 15–20 min. The seed oil served as the internal oil phase (O) at concentrations of 5% and 10% (v/v). The external aqueous phase was composed of NaCas (5% w/v) and pectin (0.1% w/v) dissolved in Millipore water, adjusted to pH 7.0.

### Characterization of nanoemulsion via DLS

The mean particle size (Z-average), poly dispersity index (PDI), and zeta-potential of the date oil nanoemulsion was measured using dynamic light scattering method (Malvern Instruments Ltd., U.K). Zeta potential, the electrical charge on the emulsion droplets, was determined under holder temperature of 25 °C and electric voltage 3.9 V by the same instrument. Diluted samples (1:50) were used for the experiments to avoid multiple scattering.

### Determination of encapsulation efficiency (EE %)

The encapsulation efficiency of nanoemulsion was determined by combining two methods with slight modifications: solvent extraction followed by the estimation of total phenolic content using Folin-Ciocalteu’s reagent (Zheng and Wang 2001). EE values are based on phenolic content rather than total oil encapsulation. Hexane and nanoemulsion (1:1 v/v) were mixed for 3 to 5 min to facilitate the solubilization of loosely bound or free date oil in hexane and then kept for 10 min for layer separation. Two layers were separated, the upper layer with free date oil in hexane and the lower layer with encapsulated-date oil. The upper hexane layer with free date oil was collected to calculate the encapsulation efficiency by measuring the total phenolic content present in the sample after removal of lower layer. Total phenolic content of freshly prepared nanoemulsion and upper layer of hexane extracted with solvent extraction was analyzed by Folin-Ciocalteu’s method given by Zheng and Wang 2001. Encapsulation efficiency was calculated as follows:

$$EE\;(\%)=\frac{Actual\;polyphenol\;content\;in\;nanoemulsion}{Total\;polyphenol\;content\;in\;fresh\;nanoemulsion\;(g)}\times100$$

$$\begin{array}{c}APC\;in\;nanoemulsion=TPC\;in\;fresh\;nanoemulsion\\-polyphenols\;in\;upper\;hexane\;layer\end{array}$$

### Transmission electron microscopy

The internal morphology of the freshly prepared nanoemulsion was examined using transmission electron microscopy (TEM). Samples were prepared by placing a drop of the nanoemulsion suspension directly onto a copper grid and allowing it to air dry. A drop of tungsten-based negative stain was then applied to enhance contrast. Imaging was performed using a JEOL JEM-1400 Plus TEM operating at an accelerating voltage of 100 kV and a magnification of 200,000X. Preliminary characterization was also conducted at 70 kV to assess general nanoparticle structure.

## Biological activities of fruit extract, seed oil, and nanoemulsion

### Antioxidant activity

The antioxidant activity of fruit extract, seed oil, and nanoemulsion was evaluated using the DPPH (1-diphenyl-2-picrylhydrazyl) radical scavenging method (Lee et al. 2010). Briefly, 500 µl of ethanolic DPPH solution (0.4 mmol) was vigorously mixed with 500 µl of fruit extract, seed oil, nanoemulsion, or water as a control and incubated at 37 °C for 1 h in the dark. Mixtures absorbance was measured spectrophotometrically at 517 nm. The scavenging activity was calculated as following:

$$\%\;Scavenging\;activity=1-\left(\frac{A_s-A_b}{A_c}\right)\ast100$$

Whereas: A_b_, A_c_, and A_s_ are the absorbance of the blank (ethanol and sample), the control (DPPH and deionized water), and the sample (DPPH and sample), respectively.

Ascorbic acid was used as a positive control while MRS medium without bacteria was used as a negative control.

ABTS radical cation scavenging assay was also used to confirm the antioxidant activity of samples. This assay based on the ability of lipid fraction in the product to scavenge 2,2’-azino-bis (3-ethylbenzothiazoline-6-sulfonic) acid (ABTS) radical cation in comparison to a standard (tocopherol -vitamin E- at a concentration of 0.1%) **(**Pannala et al. 2001). The photometric assay was conducted by mixing 0.9 ml of ABTS solution and 0.1 ml of sample for 30 min incubation in the dark. Measurements were spectrometrically taken at 734 nm. The antioxidative activity of the tested samples was calculated by determining the decrease in absorbance from the following equation:

$$\%\;Scavenging\;activity=1-\left(\frac{A_c-A_t}{A_c}\right)\ast100$$

Whereas, A_t_ and A_c_ are the respective absorbance of tested samples and ABTS.

### Antiviral activity using MTT cytotoxicity assay

To assess the half maximal cytotoxic concentration (CC_50_), stock solutions of the tested compounds were prepared in 10% DMSO in ddH_2_O and diluted further to the working solutions with DMEM. The cytotoxic activity of the sample was tested in Vero-E6 cells by using the 3-(4, 5-dimethylthiazol − 2-yl)-2, 5-diphenyltetrazolium bromide (MTT) method with minor modifications. Briefly, the cells were seeded in 96-well plates (100 µl/well at a density of 3 × 10^5^ cells/ml) and incubated for 24 h at 37 °C in 5% CO_2_. After 24 h, cells were treated with various concentrations of the tested compounds in triplicates. After 24 h, the supernatant was discarded, and cell monolayers were washed with sterile 1x phosphate buffer saline (PBS) 3 times, and MTT solution (20 µl of 5 mg/ml stock solution) was added to each well and incubated at 37 °C for 4 h followed by medium aspiration. In each well, the formed formazan crystals were dissolved with 200 µl of acidified isopropanol (0.04 M HCl in absolute isopropanol = 0.073 ml HCL in 50 ml isopropanol). Absorbance of formazan solutions was measured at λmax 540 nm with 620 nm as a reference wavelength using a multi-well plate reader. The percentage of cytotoxicity compared to the untreated cells was determined with the following equation.

The plot of % cytotoxicity versus sample concentration was used to calculate the concentration which exhibited 50% cytotoxicity (TC_50_) (Mosmann 1983).

$$\begin{array}{c}\%\;cytotoxicity\;=\\\frac{\left(absorbance\;of\;cells\;without\;treatment\;-\;absorbance\;of\;cells\;after\;treatment\right)\times100}{absorbance\;of\;cells\;without\;treatment}\end{array}$$

### Inhibitory concentration (IC_50_) determination

The IC_50_ was performed as previously described (Mostafa et al. 2020; Mahmoud et al. 2021). In 96-well tissue culture plates, 2.4 × 10^4^ Vero-E6 cells were distributed in each well and incubated overnight in a humidified 37 °C incubator under 5% CO_2_ condition. The cell monolayers were then washed once with 1x PBS and subjected to NRC-03-nhCoV virus (Kandeil et al. 2020) adsorption for 1 h at RT. The cell monolayers were further overlaid with 50 µl of DMEM containing varying concentrations of the selected test sample. Following incubation at 37 °C in 5% CO_2_ incubator for 72 h, the cells were fixed with 100 µl of 4% paraformaldehyde for 20 min and stained with 0.1% crystal violet in distilled water for 15 min at RT. The crystal violet dye was then dissolved using 100 µl absolute methanol per well and the optical density of the color measured at 570 nm using Anthos Zenyth 200rt plate reader (Anthos Labtec Instruments, Heerhugowaard, Netherlands). The IC_50_ of the compound is the concentration required to reduce the virus-induced cytopathic effect (CPE) by 50%, relative to the virus control.

## Antitumor activity and cytotoxicity

### Cell culture

PaCa-2 (Human Pancreatic Cancer cell line) and HOS (Human Osteosarcoma cell line) were maintained in DMEM media. HCT- 116 (Human Colorectal Carcinoma cell line) was maintained in DMEM media supplemented with 5% L-glutamine and RPE1 (Retinal Pigmented Epithelial normal cells) was maintained in DMEM-F12 medium. All media were supplemented with 10% fetal bovine serum and 5% antibiotic/antimycotic (penicillin/streptomycin amphotericin B) and incubated at 37 °C in 5% CO_2_ and 95% humidity.

### Cell viability assay (MTT assay)

After 24 h of seeding 50,000 cells per well in case of RPE1 (normal cell line) and 20,000 cells per well in case of HCT-116, PaCa-2 and HOS cell lines (in 96 well plates), cells were treated with different concentrations of fruit extract, seed oil, and oil nanoemulsion, dissolved in DMSO. Doxorubicin was used as a known anticancer agent (positive control). Treatments were done in triplicates and the viability of the cells was measured after 72 h of treatment. Cell viability was determined using the MTT (3-(4,5-dimethylthiazol-2-yl)-2,5-diphenyltetrazolium bromide) assay as described by Mosmann (1983**)**. The absorbance of the sample was measured at 595 nm with reference 690 nm **(**El-Hallouty et al. 2015). All measurements were normalized to untreated control.

## In vivo antimicrobial activity

### Ethical declaration

The study was designed following the ARRIVE guidelines and carried out in accordance with the U.K. Animals (Scientific Procedures) Act, 1986 and associated guidelines, EU Directive 2010/63/EU for animal experiments. All protocols and procedures that followed in this study were approved by the Animal Ethics Committee of the National Research Center (NRC), Cairo, Egypt (Approval number: 24411122021).

### Experimental birds

Throughout the experiment, the birds were housed in individually cleaned and sanitized housing and given food and water *ad libitum*. All the necessary vaccinations were given to the birds at the appropriate time (Hitchiner B1 + IB at 3 days, Gumboro at 10 days, and LaSota NDV intraocularly at 7 and 21 days). The birds were raised on a concrete floor using a deep letter system and bedding made of fresh wood shavings that were about 10 cm thick. Throughout the observation period, the chicks were kept in a constant-light system with ideal humidity, temperature, and ventilation levels. The birds were given fresh, clean water at will and a balanced diet included starter, grower, and finisher rations without any additives.

The treated birds were supplied with 1 ml containing 30 mg fruit extract/ 100 g bird/ day or 500 µl oil nanoemulsion/ bird/ day via oral gavage for 5 successive days post the experimental infection and during the treatment period.

The challenged bacterial strain’s antibiotic sensitivity test results were used to determine which antibiotics to use. Three days prior to the onset of clinical symptoms and mortality, the birds in antibiotic treated group were given 100 mg/L of 20% doxycycline in their drinking water for 5 days in a row (equivalent to approximately 10–20 mg active doxycycline/kg bodyweight/day depending on bird weight and water intake).

### Experiment 1 for *E. coli* challenge

Eighty, one day old male Cobb broiler chicks obtained from commercial Poultry Company, Egypt were used in this experiment, 5 birds at the day of arrival were sacrificed and the internal organs (yolk sac, spleen, liver, and heart blood) were cultured on nutrient broth then Congo-red media to ensure absence of pathogenic *E. coli*. Then the remaining 75 birds were randomly divided into 5 different experimental groups with 15 birds each as follow; G1E) control negative birds; G2E) control positive birds challenged with *E. coli* O78; G3E) infected birds treated with fruit extract; G4E) infected birds treated with seed oil nanoemulsion; G5E) infected birds treated with antibiotic infected birds treated with antibiotic (100 mg/L of 20% doxycycline in their drinking water for 5 days in a row).

### *E. coli* challenge strain

The microbiology department of Cairo University’s Faculty of Veterinary Medicine generously donated the *E. coli* O78 used for the experimental infection, the strain was isolated from infected birds and fully molecularly defined as reported in (Yousef et al. 2023). The strain was maintained as glycerol stocks at − 80 °C and subcultured on MacConkey agar prior to use. For challenge preparation, *E. coli* was grown overnight in LB broth at 37 °C with shaking, and bacterial suspensions were adjusted to ~ 10⁸ CFU/mL using McFarland standards. Every chicken in the different infected groups was given an oral inoculation at 2 weeks of age with 1 ml of saline containing 10^8^ fresh colony forming units (CFU) of *E. coli*/bird for two continuous days in a row.

### Experiment 2 for *C. perfringens* challenge

Seventy-five, one day old male Cobb broiler chicks obtained from commercial Poultry Company, Egypt were randomly divided into 5 different experimental groups with 15 birds each as follows; G1C) control negative birds; G2C) control positive birds challenged with *C. perfringens*; G3C) infected birds treated with fruit extract; G4C) infected birds treated with seed oil nanoemulsion; G5C) infected birds treated with antibiotic (100 mg/L of 20% doxycycline in their drinking water for 5 days in a row).

### *C. perfringens* challenge strain

The microbiology department of Cairo University’s Faculty of Veterinary Medicine generously donated the *C. perfringens* type A used for the experimental infection, it was molecularly identified and used in a previous challenge as mentioned by Salem et al. (2021). The strain was maintained as glycerol stocks at − 80 °C and subcultured on blood agar prior to use. For challenge preparation, *C. perfringens* was cultured anaerobically in fluid thioglycolate broth, and suspensions were adjusted to ~ 108 CFU/mL for inoculation. Every chicken in the different infected groups was given an oral inoculation via crop gavage at 2 weeks of age with 1 ml of saline containing 10^8^ fresh colony forming units (CFU) of 18 h freshly prepared *C. perfringens* in PBS /bird for two continuous days in a row.

### Clinical signs and mortalities

Post the experimental infection, all chicks were noticed daily for clinical signs as well as mortalities during the experimental period (28-days). Dead birds were subjected to postmortem examinations.

### *E. coli* caecal and intestinal counts

From 3 birds per group after 7 days post infection, 0.2 gm of intestinal and caecal contents were taken from each bird. These were serially diluted in sterile PBS to 1:100, 1:1000, and 1:10000, with 0.1 ml of each dilution being poured on the surface of Eosin Methylene Blue (EMB), Oxoid; UK agar plates. The plates were then incubated for 24 h at 37 °C. Typical *E. coli* colonies (metallic green) on EMB agar were counted and reported as colony-forming units (CFU) per gm.

### *C. perfringens* caecal and intestinal counts

From 3 birds per group after 7 days post infection, 0.2 g of intestinal and caecal contents were extracted from each bird and serially diluted in sterile PBS to 1:100, 1:1000, and 1:10000. 0.1 ml of each dilution was then applied to the surface of sheep blood agar plates and tryptose sulfite-cycloserine (TSC) agar supplemented with D-cycloserine and egg yolk emulsion. The plates were then incubated anaerobically for 24 h at 37 °C. Typical colonies of *C. perfringens* (black colonies) on TSC agar or large dome-shaped colonies with a double zone of haemolysis on blood agar plates were counted and reported as colony-forming units (CFU) per gm.

## Blood parameters

To measure blood parameters, blood samples were taken from the jugular vein of 3 birds per group on tubes treated with ethylene diamine tetra-acetic acid (EDTA) 24 h after treatments. Complete red blood cell count (RBCs), haemoglobin, and other blood parameters were tested. White blood cells (WBCs) or total leukocytic count (TLC), haemoglobin (Hb), haematocrit value, and differential leukocyte counts (basophils, eosinophils, heterophils, lymphocytes, and monocytes) were all investigated. Hemocytometric technique was used to obtain the complete blood picture (CBC), spectrophotometry was used to determine the Hb concentration, and Dia-Quick stained blood smears were used to determine the differential leukocyte count.

## Statistical analysis

All experiments were conducted in triplicates (*n* = 3) unless otherwise stated; all values were expressed as mean ± standard deviation and compared to the control. Survival analysis of mortality data was performed using Kaplan–Meier survival curves with confidence intervals (CIs), and differences between groups were compared using the log-rank (Mantel–Cox) test and statistical significance was considered at *p* < 0.05. All statistical analyses and visualization were performed using GraphPad Prism 8 software.

## Results and discussion

### Fatty acid composition of seed oil (GC-FID)

The fatty acid composition of *B. aegyptiaca* seed oil is an area of interest within nutritional biochemistry and plant-derived oil applications. The oil extracted from date seeds has been analyzed using gas chromatography-mass spectrometry (GC-FID) techniques, confirming the presence and relative abundance of various fatty acids. Specifically, the findings highlight the distributions of palmitic acid (C16:0), palmitoleic acid (C16:1), stearic acid (C18:0), oleic acid (C18:1n9), linoleic acid (C18:2n6), linolenic acid (C18:3n3), arachidic acid (C20:0), gadoleic acid (C20:1), and behenic acid (C22:0) within the oil matrix. Oleic acid (C18:1n9) constitutes a significant portion of the fatty acid profile at approximately 42%, followed by linoleic acid (C18:2n6) at around 32%. These monounsaturated and polyunsaturated fatty acids represent over 75% of the total fatty acid content, underscoring the oil’s potential as a dietary source of essential fatty acids. The health implications of oleic acid are well-documented, and it is known for its role in promoting heart health and improving insulin sensitivity (Sagne et al. 2019; Bazongo et al. 2024). The presence of saturated fatty acids such as palmitic acid and stearic acid, at approximately 12% and 7%, respectively, indicates that while date seed oil does provide some saturated fats, the favorable ratio of unsaturated to saturated fatty acids suggests a healthful profile that supports its use in food and cosmetic applications (Chomini et al. 2020; Aremu et al. 2022). Additionally, several studies indicated that this oil has low moisture content, enhancing oxidative stability and prolonging shelf life, improving usability in various formulations (Zang et al. 2018; Chomini et al. 2020). Although linolenic acid (C18:3n3) is present in lower amounts (about 2%), its inclusion suggests potential anti-inflammatory effects, contributing to the diversity of beneficial fatty acids in the oil (Etigany et al. 2023; El Kaourat et al. 2024). Oils rich in omega-3 fatty acids can serve as significant supplements, supporting the health claims of date seed oil (Keroletswe et al. 2017). Minor components like arachidic acid, gadoleic acid, and behenic acid also enhance the oil’s overall quality and usability, making it suitable for both culinary and industrial applications. These fatty acids can improve the functional properties of products derived from this oil, contributing positively to the texture and sensory attributes of skincare items and biodiesel formulations (Jauro and Adams 2011; Lerma-García et al. 2015). Collectively, these fatty acids provide various health benefits, advocating for the use of *B. aegyptiaca* seed oil as a viable alternative to more commonly used oils, particularly in regions where it is indigenous. The high oleic and linoleic acid content positions it favorably in the global market for healthy cooking oils, increasing food safety and nutritional value (El harkaoui et al. 2023; Etigany et al. 2023). Moreover, studies have noted the presence of antioxidants in these oils, further contributing to their health benefits and applications (Jauro and Adams 2011; Altuna et al. 2018).

In conclusion, the comprehensive analysis of seed oil’s fatty acid composition highlights its nutritional profile (Table 1). It suggests avenues for further exploration into its applications across various sectors, including food science, dietary supplements, and cosmetics. The favorable unsaturated to saturated fat ratio, combined with bioactive compounds, underscores its potential as a valuable resource in promoting health and well-being.

**Table 1:**
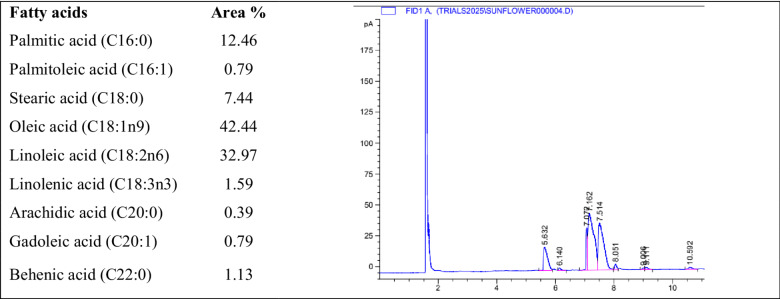
GC-FID analysis of date seed oil identity

### Physicochemical characteristics of sodium caseinate

Sodium caseinate (NaCas), a milk-derived protein primarily obtained from cow’s milk, exhibits unique physicochemical properties that make it a valuable ingredient in various food applications. Table 2 presents the physicochemical profile of a cow-derived NaCas sample, highlighting key parameters: moisture content (3.04 ± 0.10%) and protein content (90.51 ± 0.02%), both aligning with industrial standards (protein > 88%) (Mehra et al. 1998). The lactose content (0.91 ± 0.03%) remains below 1%, typical of post-processing levels and beneficial in reducing Maillard reaction risks during thermal processing. Additional parameters include ash (4.28 ± 0.05%), fat (1.07 ± 0.02%), and colorimetric values—*lightness (L: 12.29 ± 0.02), *redness (a: 1.70 ± 0.03), and *yellowness (b: 11.04 ± 0.05).

**Table 2:** Physicochemical characteristics of sodium caseinate

Parameters	Value
Moisture (%)	3.04 ± 0.10
Protein (%)	90.51 ± 0.02
Lactose (%)	0.91 ± 0.03
Ash (%)	4.28 ± 0.05
Fat (%)	1.07 ± 0.02
**Color**	
Lightness (L^*^)	12.29 ± 0.02
Redness (a^*^)	1.70 ± 0.03
Yellowness (b^*^)	11.04 ± 0.05

The low moisture content (~ 3.04%) enhances the stability and shelf life of NaCas, making it suitable for long-term storage in food systems by inhibiting microbial growth (Freitas et al. 2017). Its high protein content (90.51%) not only provides nutritional enrichment but also contributes functional benefits, particularly in emulsification, improving texture and stability in emulsified food products (Bojanic-Rasovic et al. 2013; Bonilla and Sobral 2017).

The minimal lactose level (0.91%) renders NaCas appropriate for lactose-intolerant consumers and reduces the risk of undesirable fermentation during storage (Morales-Irigoyen et al. 2012). Moreover, the trace lactose can still participate in modifying the rheological and textural properties of dairy-based products (Rajanna et al. 2024).

The ash content (4.28%) reflects the presence of minerals that enhance both nutritional and functional attributes. These minerals aid in stabilizing emulsions and improving textural properties, which can influence flavor and mouthfeel (Goswami et al. 2019, 2021). Meanwhile, the low fat content (1.07%) supports the inclusion of NaCas in low-fat or calorie-reduced formulations. Despite the reduced fat, NaCas maintains key techno-functional roles, even serving as an effective fat replacer in healthier product development (Wang et al. 2016).

Color properties also play a crucial role in consumer perception. The lightness value (L^*^: 12.29) indicates a pale cream hue typical of milk proteins, while a^*^ and b^*^ values provide color stability benchmarks. These parameters are critical for assessing product consistency and acceptance, especially in applications where appearance influences consumer choice (Ayyash and Shah 2010; Henneberry et al. 2015).

In summary, the physicochemical attributes of cow-derived sodium caseinate demonstrate its excellent potential as an emulsifier for date oil, due to its nutritional profile, functional performance, and stability in complex food systems.

### Characterizations of date oil nanoemulsion

Nanoemulsions are colloidal dispersions that consist of submicron-sized droplets (typically between 20 and 600 nm) of one liquid phase dispersed within another immiscible liquid, often stabilized by surfactants. Table 3 summarizes the physicochemical properties of the produced nanoemulsion, including droplet size, polydispersity index (PDI), zeta potential, and encapsulation efficiency (EE %). The average droplet size of NaCas nanoparticles (NaCas-Pec NPs) is measured at 115.45 ± 4.1 nm. When 5% date oil is incorporated into the formulation, the droplet size increases to 130.50 ± 5.2 nm, and at 10% date oil concentration, the droplet size further enlarged to 152.90 ± 7.4 nm. The increase in droplet size with higher oil concentrations can be attributed to the oil phase disrupting the stability of the emulsion, resulting in larger aggregate sizes **(**Nirmala and Nagarajan 2017). This phenomenon has been observed in other studies, where the addition of oil led to a significant increase in droplet dimensions, affecting the overall stability of the nanoemulsion (Weerapol et al. 2022).

**Table 3:** Physicochemical properties of seed oil nanoemulsion

Sample	Size	PDI	Zeta	EE (%)
NaCas-Pec NPs	115.45 ± 4.1	0.175	-37.10 ± 5.7	-
5% seed oil	130.50 ± 5.2	0.180	-43.80 ± 8.6	92.12 ± 2.23
10% seed oil	152.90 ± 7.4	0.368	-38.00 ± 7.9	86.76 ± 3.21

NaCas-Pec NPs: 5% sodium caseinate and 0.1% pectin stirred and sonicated as control nanoemulsion; 5% seed oil: 5% seed oil, 5% sodium caseinate, and 0.1% pectin stirred and sonicated; 10% seed oil: 10% seed oil, 5% sodium caseinate, and 0.1% pectin stirred and sonicated. EE (%): encapsulation efficiency %.

The PDI values, indicating the distribution of droplet sizes, were recorded as 0.175, 0.180, and 0.368 for NaCas NPs, 5%, and 10% seed oil, respectively. A lower PDI signifies a more homogeneous emulsion; hence, the PDI values suggested that the formulations with 5% seed oil maintain better droplet size uniformity than those with 10% concentration (Anjali et al. 2012). This increase in PDI with higher oil content potentially suggests the aggregation phenomena, where larger droplets coalesce, leading to an unstable emulsion system (Weerapol et al. 2022).

Zeta potential measurements reflect the charge of the nanoemulsion droplets, a critical factor for predicting their stability. The zeta potential for NaCas NPs was − 37.10 ± 5.7 mV, which became more negative at -43.80 ± 8.6 mV for the 5% seed oil formulation, while the 10% seed oil formulation resulted in a zeta potential of -38.00 ± 7.9 mV. A high negative charge indicates good electrostatic stabilization for the nanoemulsions, which is essential for preventing droplet coalescence (Oliyaei et al. 2022). The enhanced negative zeta potential in the 5% seed oil emulsion implies stronger repulsive forces between droplets, contributing to its stability compared to the 10% oil formulation.

Sodium caseinate (NaCas) can provide good stability for nanoemulsions for periods exceeding one month, with some studies showing stability for at least six months, particularly when the droplets are well below 200 nm (Yerramilli and Ghosh 2017).

The stability of nanoemulsions is influenced by various physicochemical properties, including particle size, polydispersity index (PDI), zeta potential, and encapsulation efficiency (EE). Firstly, results in Table 3 indicated that particles size maintained under 200 nm which generally indicates a stable emulsion system (Ahmad et al. 2023). Moreover, the PDI below 0.3 is indicative of a narrow particle size distribution, which is desirable for better stability against phenomena such as Ostwald ripening and droplet coalescence (Agnish et al. 2022). The negative charges suggest that the emulsions are likely to remain stable under varying environmental conditions, thereby prolonging their shelf life and functionality (Sreeharsha et al. 2021). These findings resonate with the literature, which asserts that high zeta potential values enhance the stability of nanoemulsions by minimizing the likelihood of flocculation or aggregation (Hwang et al. 2024).

Encapsulation efficiency (EE) is a key performance metric, particularly for applications requiring effective delivery of bioactive compounds. The EE for the 5% seed oil nanoemulsion is exceptionally high as 92.12 ± 2.23%, compared to 86.76 ± 3.21% for the 10% concentration. The high encapsulation efficiency indicates that a significant proportion of the date oil’s active compounds can be effectively retained within the emulsion matrix, contributing to their stability and potential bioactivity when delivered in food or nutraceutical applications (Fernandes et al. 2021).

## Transmission electron microscopy of nanoemulsions

Nanoemulsions, characterized by their small droplet sizes, have attracted significant attention in various fields, including food science, pharmaceuticals, and cosmetics. This interest is due, in part, to their ability to enhance the bioavailability of lipophilic compounds. Transmission Electron Microscopy (TEM) is a crucial tool for visualizing the morphology and size distribution of these nanoemulsions at the nanoscale, providing valuable insights into their structural characteristics.

**Fig. 1:**
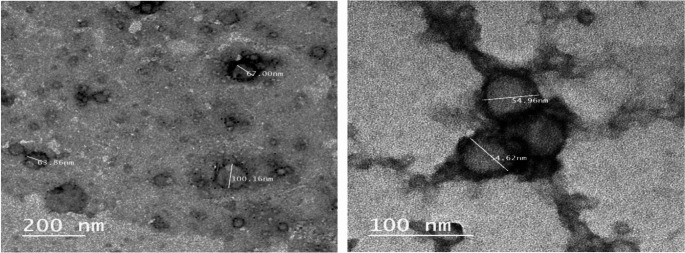
TEM micrograph of seed oil nanoemulsion, magnification of 200,000X

Figure 1 shows the morphology of seed oil nanoemulsions formulated with sodium caseinate (NaCas) and pectin via TEM. Studies have shown that the size and shape of nanoparticles can directly influence their stability and functional properties (Arredondo-Ochoa et al. 2017; Radwan et al. 2023). In the present study, nanoemulsions created with 5% and 10% oil exhibited spherical shapes in TEM images. For instance, Radwan et al. reported that droplet sizes of oil-based nanoemulsions typically appear as spherical structures under TEM, corroborating findings from dynamic light scattering (DLS) measurements (Radwan et al. 2023). In our observations, droplet sizes for both 5% and 10% oil nanoemulsions were confirmed to be consistent with the sizes previously determined through DLS, demonstrating the integrity of the size distribution analysis provided by TEM.

### Biological activity of fruit extract, seed oil, and nanoemulsion

#### Antioxidant activity

**Fig. 2:**
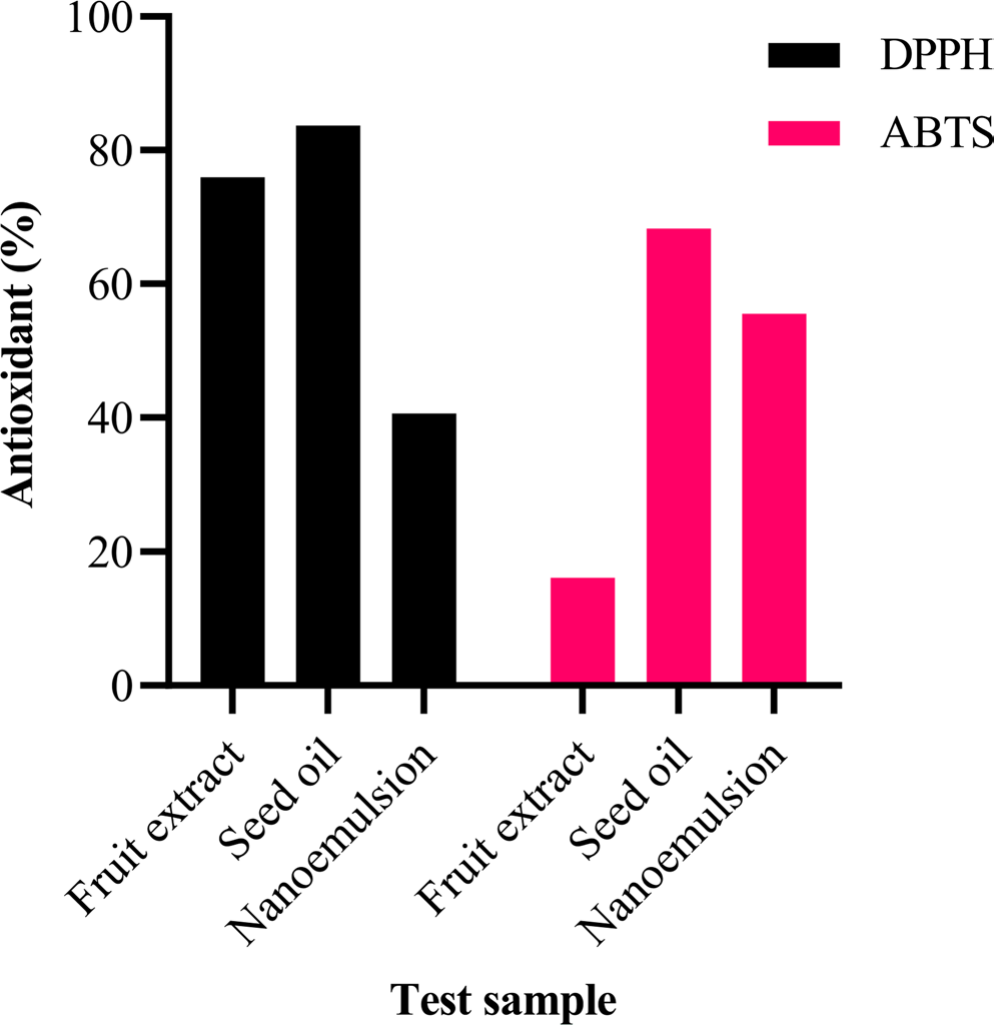
Antioxidant activity of fruit extract, seed oil, and nanoemulsion

*B. aegyptiaca* is a medicinal plant rich in bioactive compounds with antimicrobial, antioxidant, and anti-inflammatory properties (Hassan et al. 2016). Recent advancements in nanotechnology have reserved its therapeutic potential through nano-preparation methods (Salem et al. 2021). The obtained data demonstrated that fruit extract exhibits notable antioxidant properties (Fig. 2). Antioxidant assays (DPPH and ABTS) revealed that the seed oil had the strongest radical scavenging activity, followed by the fruit extract, while the nanoemulsion displayed reduced antioxidant potential. This may indicate that nano-formulation impacts the availability of bioactive compounds.

#### Antiviral activity

**Fig. 3:**
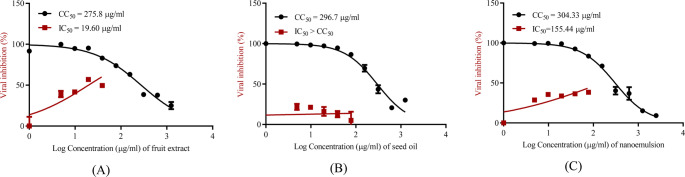
Anti-SARS-CoV-2 virus activity of (**A**) fruit extract, (**B**) seed oil, and (**C**) nanoemulsion

The fruit extract demonstrated notable antiviral activity against SARS-CoV-2 in VERO E6 cells, with an IC₅₀ of 19.60 µg/mL and a CC₅₀ of 275.8 µg/mL (Fig. 3), resulting in a selectivity index (SI) of 14. As the CC₅₀ value substantially exceeds the IC₅₀ (CC₅₀ > IC₅₀; SI > 1), this suggests a promising therapeutic potential of the fruit extract against SARS-CoV-2. In contrast, the oil nanoemulsion exhibited lower antiviral efficacy, with an IC₅₀ of 155.44 µg/mL and a CC₅₀ of 304.33 µg/mL, yielding an SI of only 1.9, which indicates limited antiviral potency. Meanwhile, the seed oil showed no effective antiviral activity, as the IC₅₀ exceeded the CC₅₀, implying cytotoxicity at concentrations required to inhibit viral replication. A 2010 study investigated the fixed oil extracted from *B. aegyptiaca* fruit and assessed its biological activities. Preliminary screening revealed antiviral effects against *Herpes simplex* virus in vertebrate (Vero) cells, highlighting its potential as a natural antiviral agent (Al Ashaal et al. 2010).

#### Antitumor activity

Fruit extract also exhibited potent, dose-dependent cytotoxicity against cancer cell lines (HCT-116 and PaCa-2), especially at higher concentrations, whereas the seed oil and nanoemulsion were more effective against HOS cells. Importantly, the fruit extract and nanoemulsion showed selective toxicity, as both were relatively safe for normal RPE1 cells at moderate doses.

## HCT-116 (Human Colorectal Carcinoma cell line)

**Fig. 4:**
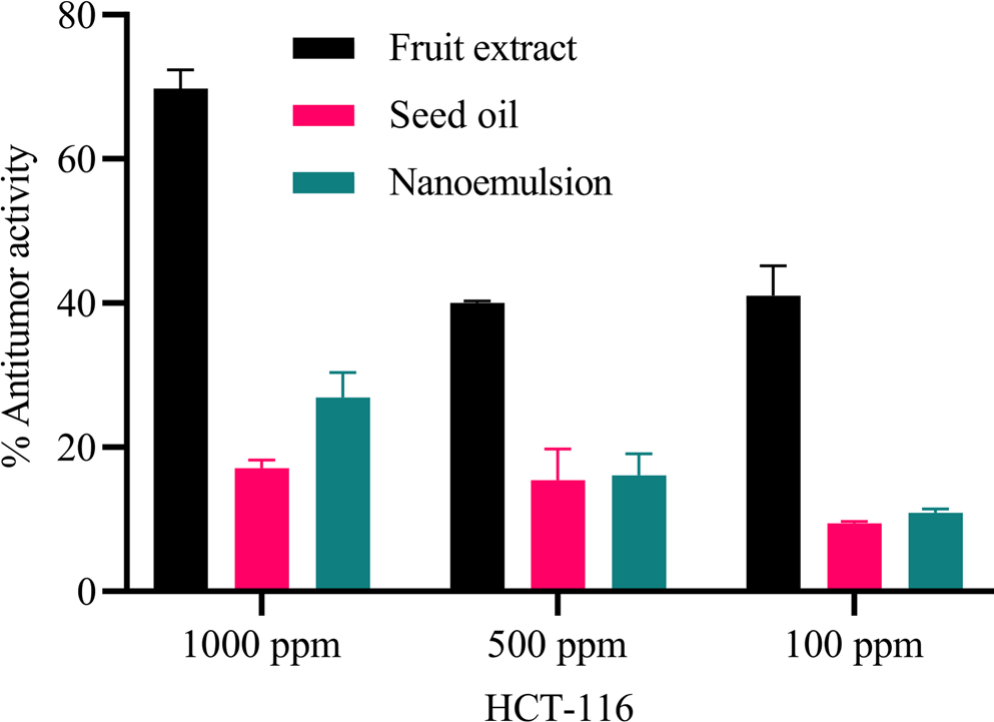
Antitumor activity of fruit extract, seed oil, and nanoemulsion against HCT-116 carcinoma

Fruit extract exerted 69.75% cytotoxicity at 1000 ppm concentration. Whereas, at 500 and 100 ppm concentrations, the cytotoxicity detected was 40% and 41% respectively. No significant cytotoxicity was detected of both the oil and its emulsion (Fig. 4).

## HOS (Human Osteosarcoma cell line)

**Fig. 5:**
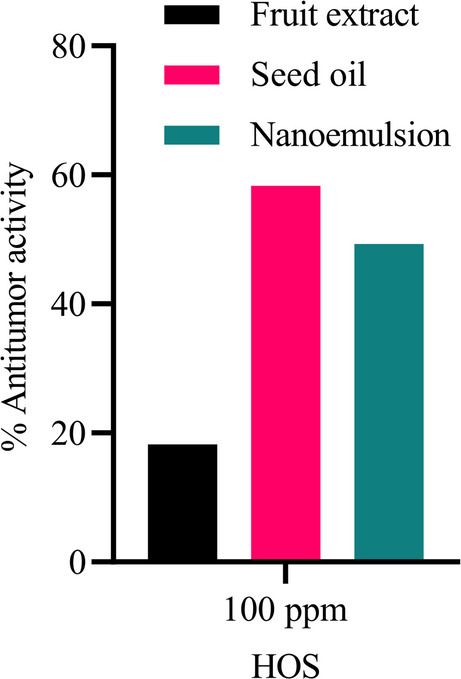
Antitumor activity of fruit extract, seed oil, and nanoemulsion against HOS

Cytotoxicity results on Osteosarcoma cell line showed that the oil and its emulsion have cytotoxicity of 49.3 and 58.3 respectively. No significant cytotoxicity was detected by fruit extract (Fig. 5).

## PaCa-2 (Human Pancreatic Cancer cell line)

**Fig. 6:**
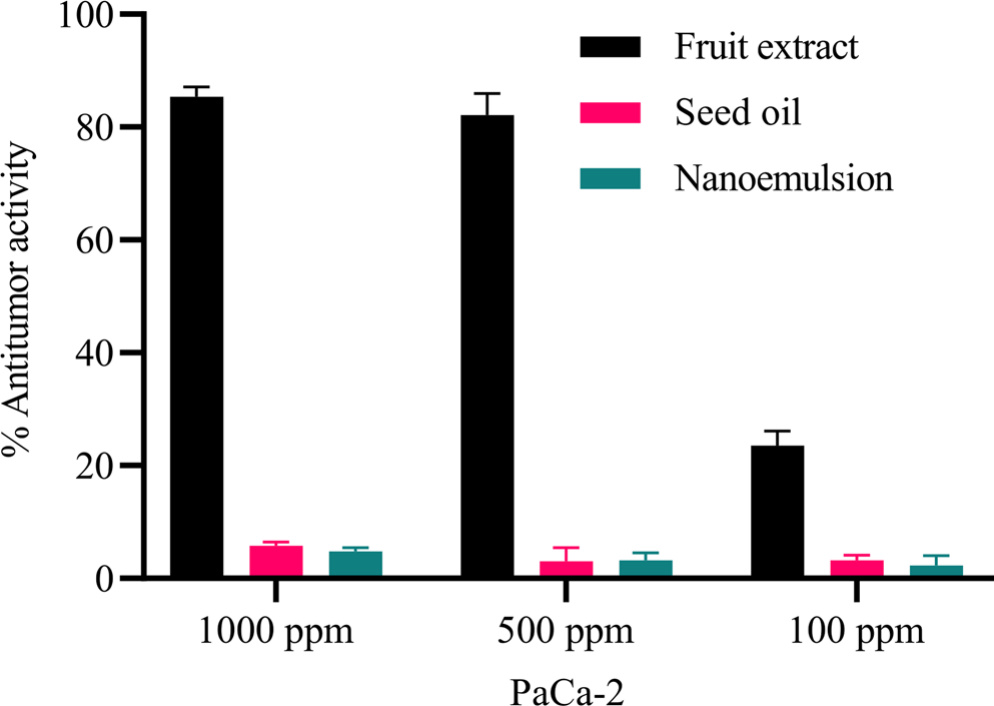
Antitumor activity of fruit extract, seed oil, and nanoemulsion against PaCa-2 carcinoma

Fruit extract was toxic to pancreatic cancer cell line at 500 and 1000 ppm concentrations (cytotoxicity 82.1% and 85.4%, respectively). No significant toxicity of the oil or its emulsion was detected (Fig. 6).

## RPE1 (Retinal Pigmented Epithelial normal cells)

**Fig. 7:**
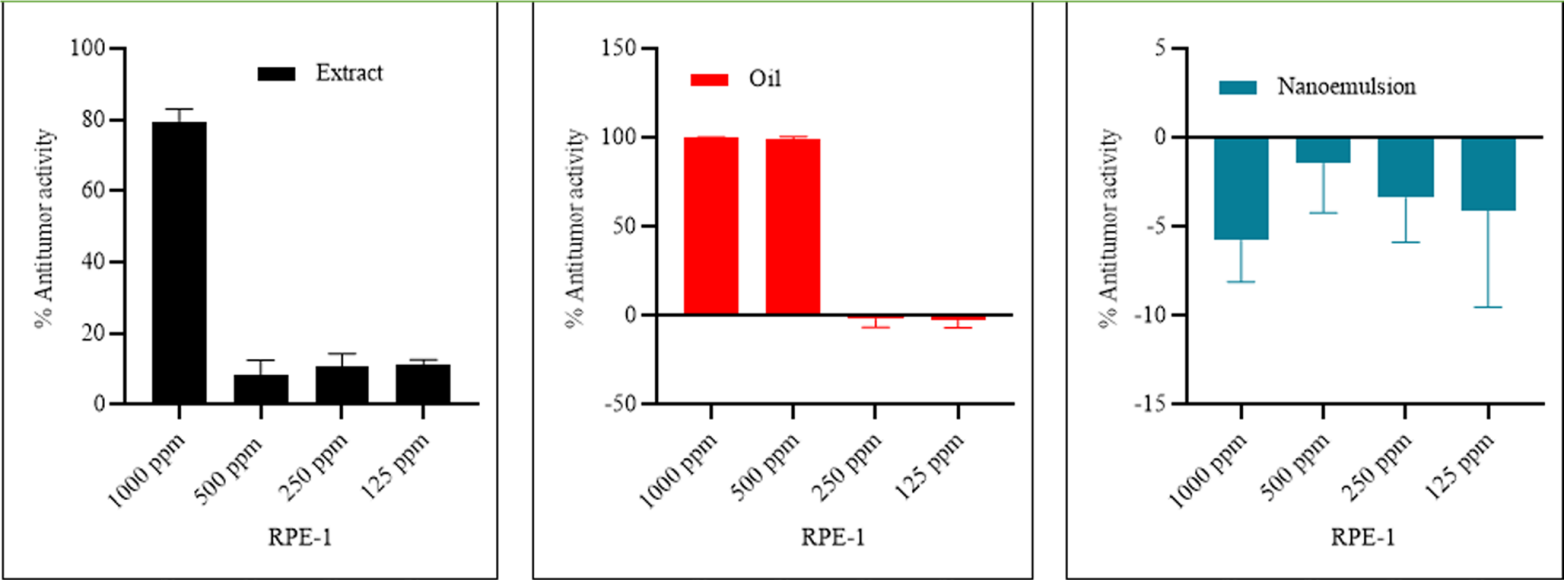
Cytoxicity of fruit extract, seed oil, and nanoemulsion against RPE-1 normal cells

Cytotoxicity test on normal cells showed that fruit extract was safe till 500 ppm concentration. The oil extract was highly toxic at 500 and 1000 ppm and safe at lower concentrations, while the nanoemulsion of oil was safe at all tested concentrations (Fig. 7).

Al Ashaal et al. 2010 stated that date oil contained 54.53% unsaturated fatty acids and 1.14% sterols. The oil exhibited anticancer activity against lung, liver and brain human carcinoma cell lines. A mixture of steroidal saponins, balanitin‑6 and balanitin‑7, isolated from its kernels exhibited potent anti-proliferative action in vitro (IC₅₀: 0.3–0.5 µM against lung and glioblastoma lines) and extended survival in a mouse leukemia model, comparable to vincristine (Gnoula et al. 2008). Methanolic fruit extract demonstrated significant cytotoxicity against human breast (MCF‑7), prostate (PC‑3), and colon (Caco‑2) cancer cell lines, with a selective induction of apoptosis and cell cycle arrest (Ibrahim et al. 2022).

In general, the observed biological activities correlated well with the presence of phytochemicals such as saponins, flavonoids, and phenolic compounds in the fruit extract, as well as fatty acids identified by GC–MS analysis. The seed oil was rich in oleic and linoleic acids, which are known for their antimicrobial, antioxidant, and anti-tumor properties.

### In vivo antimicrobial activity in infected chicks.

**Fig. 8:**
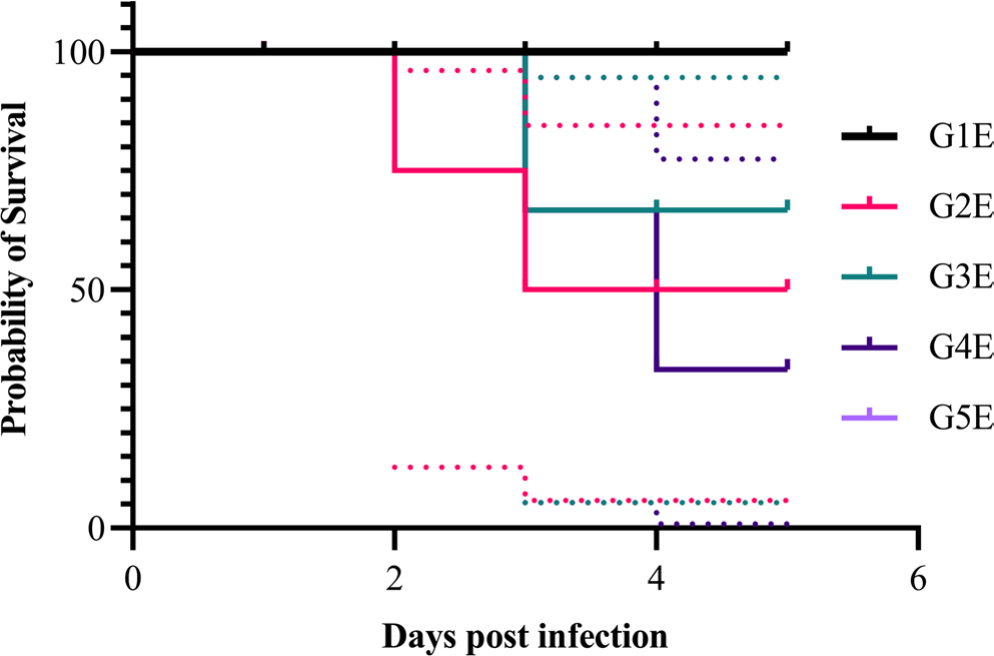
Kaplan–Meier survival plot with confidence intervals (CIs) of mortality for *E. coli* challenge

In the negative control group of experiment 1 (G1E), survival remains at 100% throughout the study validating the experimental setup. In the positive control group (G2E), a marked decline in survival (~ 67%) was observed, with a mortality rate of 33% (5 out of 15 birds), reflecting the severe impact of *E. coli* O78 challenge in untreated birds. The wide CI bands reflect greater variability in mortality timing (Fig. 8). In the fruit extract-treated group (G3E), survival is high (~ 93%) with only one death (6.6% mortality, 1/15). The curve is close to the negative control, suggesting strong protective efficacy of the fruit extract. The CI bands are narrow, reflecting consistent protection across birds. In the oil nanoemulsion-treated group (G4E), intermediate survival (~ 73% by end, 4/15 deaths) was observed, which was better than positive control but weaker than fruit extract or antibiotic. The CI overlaps partially with both positive control and fruit extract, showing moderate efficacy. In the antibiotic-treated group (G5E), survival is ~ 87% (2/15 deaths), showing good protection but slightly less effective than fruit extract in this case. CI bands are reasonably narrow, supporting reliability.

Fruit extract succeeded in reducing the mortality rate in birds challenged with *E. coli* O78. The clinical signs appeared in G2E, G3E, G4E and G5E in one day post infection in shape of brownish droppings, ruffled feathers, off food, huddling together, and un-thriftiness. The severity of clinical signs was less in both G3 and G5 when compared with G2E. G1E showed no clinical signs.

Kaplan–Meier survival analysis of *E. coli* challenge experiment (Fig. 8) revealed no significant differences in survival among groups (log-rank test: χ² = 6.264, df = 4, *p* = 0.1803). Despite some numerical variation in mortality rates between groups, the differences were not statistically significant. This suggests that either the treatment effects on survival were modest or that the sample size (*n* = 15 per group) limited statistical power to detect differences.

**Fig. 9:**
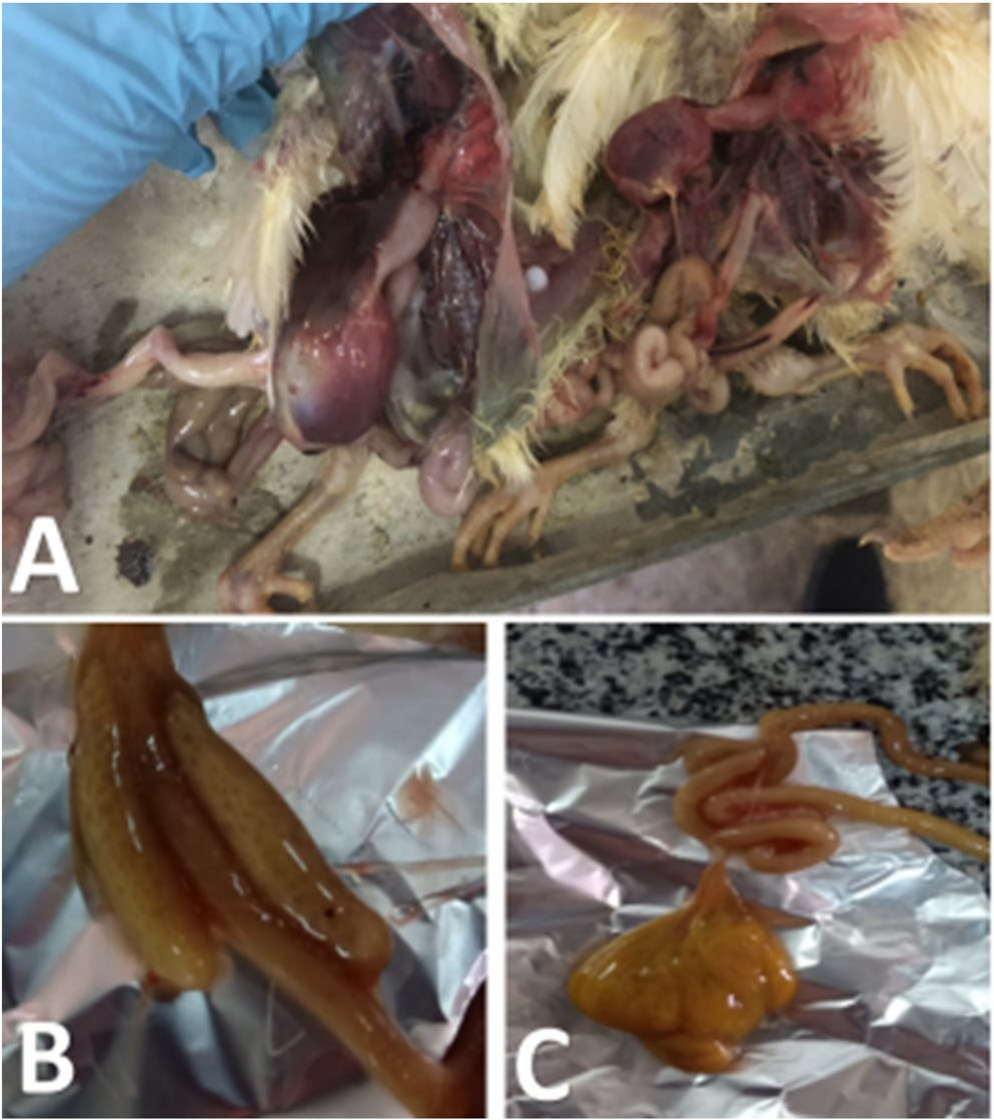
PM lesions of freshly dead birds from G2E experimentally challenged with *E. coli* O78. **A**: congested carcass with septicemia; **B**: two blind caeci filled with gases; **C**: unabsorbed yolk sac

**Fig. 10:**
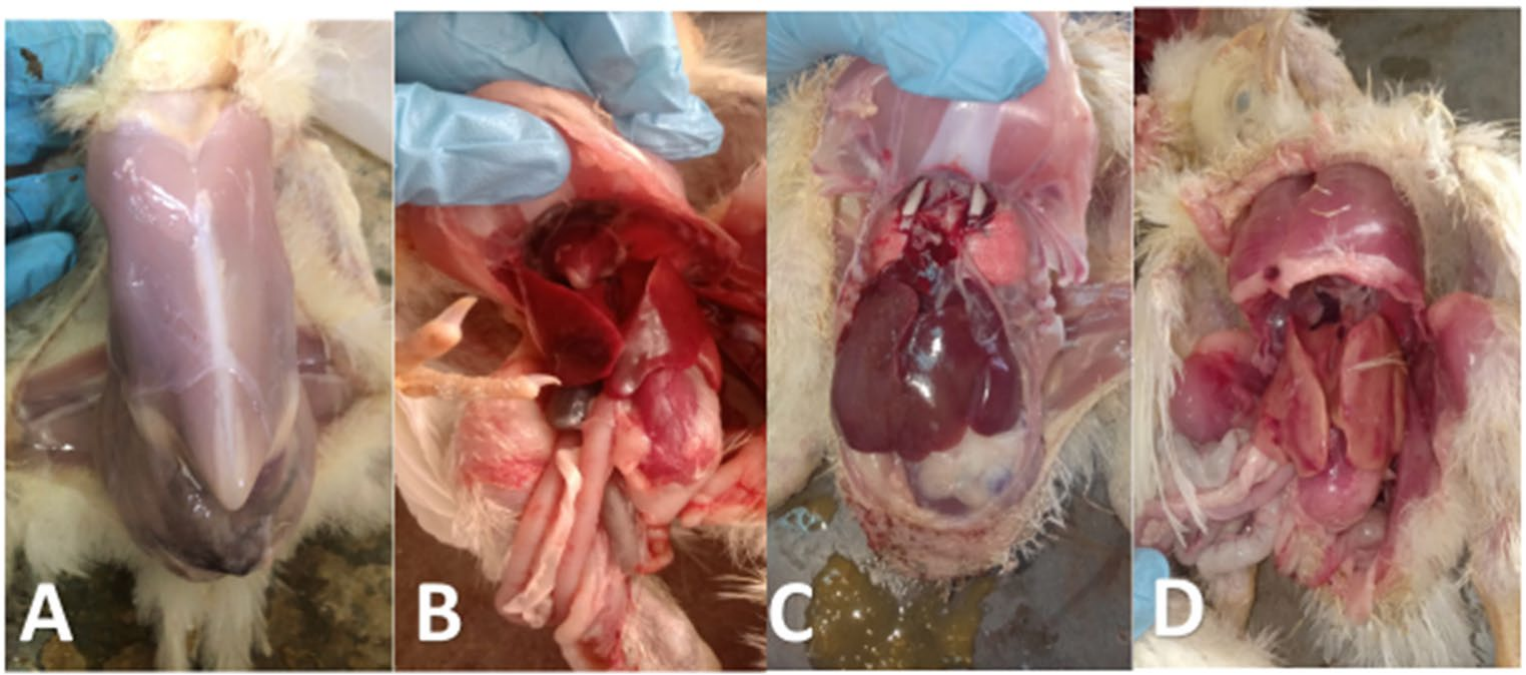
PM lesions of birds from different experimental groups. **A**: normal PM in negative control; **B**: nearly normal PM appearance of birds treated with fruit extract; **C**: brownish diarrhoea, congested liver with normal lungs in birds treated with seed oil nanoemulsion; D: subcapsular hemorrhages in the liver in birds treated with antibiotic.

As noticed in Figs. 9 and 10, the PM lesions in G2E were noticed as distended caeci with gases, enteritis, nephritis and ureters distended with ureates, congestion in the liver, pericarditis, air sacculitis, perihepatitis, congestion in muscles and subcutaneous blood vessels, and un-absorbed yolk sac. The severity of PM lesions was less in G3 and G5 than G2. G1 showed no PM lesions. G3E and G5E showed lower caecal and intestinal *E. coli* colonization when compared with G2E (Table 4). Seed oil nanoemulsion showed good but not promising results.

**Table 4:** Caecal and intestinal bacterial count

	Bacterial count log₁₀ (CFU/g) ± SEM at 7 days post infection									
	G1		G2		G3		G4		G5	
***E. coli*** **O78**	Caecal	Intestinal	Caecal	Intestinal	Caecal	Intestinal	Caecal	Intestinal	Caecal	Intestinal
	6.78 ± 0.11	7.11 ± 0.48	9.79 ± 0.85	9.46 ± 0.61	3.69 ± 0.33	3.75 ± 0.77	7.27 ± 0.49	6.72 ± 0.76	5.63 ± 1.05	3.94 ± 0.64
***C. perfringens***	8.23 ± 0.36	7.49 ± 0.35	11.56 ± 0.36	11.81 ± 0.35	4.08 ± 0.15	3.79 ± 0.60	8.08 ± 0.09	7.61 ± 0.53	4.95 ± 0.06	4.49 ± 0.34

G1E) control negative birds; G2E) control positive birds challenged with *E. coli* O78; G3E) infected birds treated with fruit extract; G4E) infected birds treated with seed oil nanoemulsion; G5E) infected birds treated with antibiotic.

The examined blood parameters revealed that no substantial differences were observed in all the treated groups (G3E, G4E and G5E) in comparison with the control negative birds (G1E), while the changes in the blood parameters were observed in G2E experimentally challenged with *E. coli* O78. The high WBCs and heterophil counts in infected groups indicate an active immune response (Table 5).

**Table 5:** Blood parameters of challenged groups versus control

Blood parameters	G1E	G2E	G3E	G4E	G5E	G1C	G2C	G3C	G4C	G5C	Reference range
RBCs	2.7	1. 6	2.2	2.1	1.9	2.9	1.9	2.3	2.1	1.88	2.5–3.5 × 10^6^ µl
Hemoglobin (Hb)	7.5	5.6	6.8	6.7	6	7.2	6.5	7.5	6.5	6.2	7–13 g/dl
Hematocrit (PCV)	22.5	16.8	20.4	20.1	18	21.6	19.5	22.5	19.5	18.6	22–35%
WBCs (TLC)	27	119	89	82	90	25	98	83	84	83	15–30 × 10^3^ /µl
	**Differential count**										
Basophils	0	0	0	0	0	0	0	0	0	0	0–2%
Eosinophils	2	2	3	2	3	2	3	2	2	3	1–4%
Heterophils	43	51	50	50	48	40	53	48	46	46	20–50%
Lymphocytes	52	34	39	38	39	55	33	41	42	40	40–75%
Monocytes	3	13	8	10	10	3	11	9	10	11	1–10%

G1E) control negative birds; G2E) control positive birds challenged with *E. coli* O78; G3E) infected birds treated with fruit extract; G4E) infected birds treated with seed oil nanoemulsion; G5E) infected birds treated with antibiotic.

**Fig. 11:**
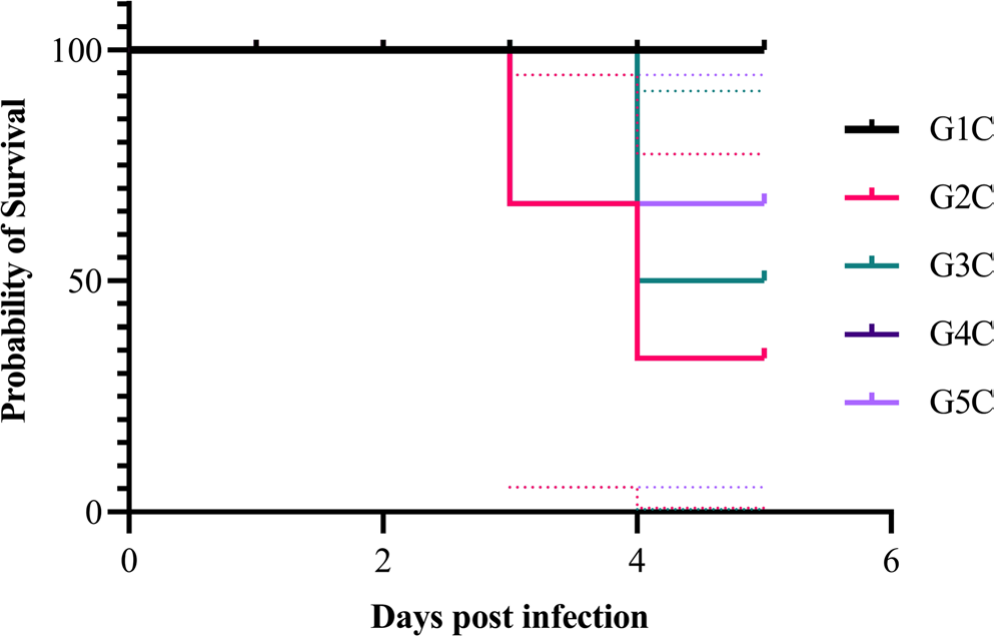
Kaplan–Meier survival plot with CIs of mortality for *C. perfringens* challenge

In the negative control group of experiment 2 (G1C), all birds survived throughout the trial (100% survival), confirming the absence of background mortality under unchallenged conditions and validating the experimental setup. In the positive control group (G2C), a moderate decline in survival to 80% (3/15 mortalities), reflecting the pathogenic effect of *C. perfringens* infection in untreated birds. The wide CI bands reflect greater variability in mortality timing (Fig. 11). In the fruit extract-treated group (G3C), survival is high (~ 93%) with only one death (6.6% mortality, 1/15), indicating a strong protective effect of fruit extract against *C. perfringens*. In the oil nanoemulsion-treated group (G4C), 13% mortality (2/15) was observed, suggesting intermediate protection, better than the positive control but slightly less effective than the fruit extract or antibiotic treatments. In the antibiotic-treated group (G5C), 6.6% mortality (1/15) was recorded, comparable to the fruit extract group, confirming the efficacy of standard antibiotic therapy. The CI bands in Fig. 11 confirmed that while all treated groups (G3C–G5C) tended to have higher survival than the positive control (G2C), the overlapping intervals indicated that the improvements were not statistically significant (*p* > 0.05) according to the log-rank test.

Fruit extract succeeds in reducing the mortality rate in birds challenged with *C. perfringens* that also outperformed the seed nanoemulsion. The clinical signs appeared in G2C, G3C, G4C, and G5C in one day post infection in shape of orange color to brownish droppings mixed with gases, off food, ruffled feathers, huddling together, and un-thriftiness. The severity of clinical signs was less in both G3C and G5C when compared with G2C. G1C showed no clinical signs.

Kaplan–Meier survival analysis showed numerical reductions in mortality in extract- and antibiotic-treated groups compared to the positive control (Fig. 11). However, these differences were not statistically significant (log-rank test: χ² = 4.403, df = 4, *p* = 0.3542). Although differences in observed mortality percentages were present (e.g., positive control 33% vs. extract-treated 6.6%), the sample size (*n* = 15/group) limited statistical power. As a result, these numerical reductions in mortality did not reach statistical significance.

**Fig. 12:**
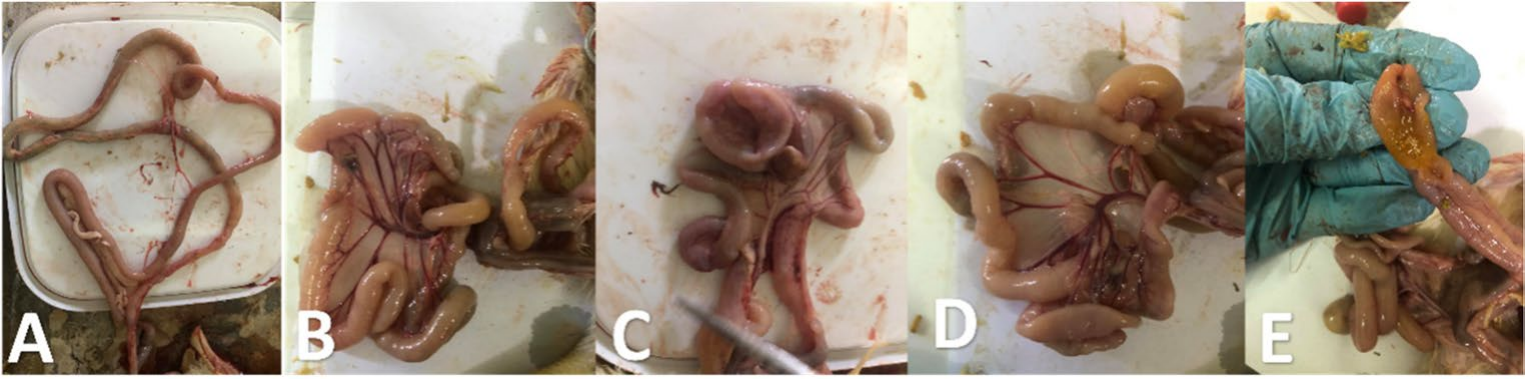
PM lesions of birds from different experimental groups. **A**: normal intestine in negative control; **B**: severe congested mesenteric blood vessels with ballooning of intestine with thin mucosa in positive control birds experimentally challenged with *C. perfringens*; **C**: less inflammation of intestine in birds treated with fruit extract; **D**: ballooning in intestine with congestion in blood vessels of birds treated with seed oil nanoemulsion; E: undigested feed particles mixed with gases with enteritis in birds treated with antibiotic.

As seen in Fig. 12, the PM lesions in G2C were noticed as distended intestine with gases, hemorrhagic patches in the intestinal mucosa, enteritis with presence of un-digested feed particles. The severity of PM lesions was less in G3C and G4C than G2C. The severity of PM lesions was less in G3C and G5C than G2C. G1C showed no PM lesions. G3C and G5C showed lower caecal and intestinal *C. perfringens* colonization when compared with G2C (Table 4).

The examined blood parameters revealed that no big differences were observed in all the treated groups (G3C, G4C, and G5C) in comparison with the control negative birds (G1C), while the changes were observed in the blood parameters in G2C experimentally challenged with *C. perfringens* (Table 5). The current results also confirm earlier findings by Negm El-Dein et al. (2024); Shakal et al. (2024b), who documented the pathogenicity of *E. coli* O78 and *C. perfringens* type A in poultry, including characteristic PM lesions and systemic involvement. In both experiments, fruit extract treated birds showed lower caecal and intestinal bacterial counts for *E. coli* and *C. perfringens*. The antibacterial effect of desert date is attributed to a variety of bioactive compounds in its different parts especially leaves, seeds, bark, and fruits (Tula et al. 2014). The antibacterial efficacy of *B. aegyptiaca* may be contributed to its richness in different potent active principles with antibacterial effect and these phytochemicals involved: saponins that cause membrane disruption; flavonoids which have antioxidant activity with enzyme inhibition; alkaloids that have DNA/protein synthesis interference and tannins precipitation of microbial proteins (Tula et al. 2014; Ezemokwe et al. 2020). In addition, the mode of action of *B. aegyptiaca* active principles antibacterial properties is not completely understood in all cases, but several mechanisms have been proposed based on research and these include: (1) disruption of bacterial cell membranes as many studies suggest that *B. aegyptiaca* extracts contain saponins, alkaloids, and phenolics, which can interact with bacterial cell membranes, causing increased permeability leading to leakage of cellular contents, cell lysis and death; (2) inhibition of protein and nucleic acid synthesis as some of the phytochemicals may interfere with bacterial enzymes involved in DNA replication or protein synthesis, thus inhibiting growth or killing the bacteria; (3) oxidative stress induction as the phenolic compounds and flavonoids in the plant may induce oxidative stress in bacterial cells by generating reactive oxygen species (ROS), leading to damage of proteins, lipids, and DNA; (4) enzyme inhibition as there’s also evidence that extracts can inhibit bacterial metabolic enzymes, disrupting essential pathways for energy production and survival (Kahsay et al. 2014; Ishaku et al. 2020).

In both experiments, birds treated with fruit extract did not show significant differences in the blood parameters. Importantly, hematological analysis indicated that neither the extract nor its nanoemulsion induced significant deviations in RBC, WBC, or differential counts. These findings confirm the extract’s safety profile, consistent with Abdullahi et al. (2020), who reported no adverse hematological effects in broilers fed *B. aegyptiaca* fruit meal. On the other hand, in the current study, birds treated with antibiotic showed little changes in blood parameters than normal. Trîncă et al. (2015) confirmed that oxytetracycline does not reveal a significant change in blood pictures of treated birds except those treated with the highest dose of the drug and contributed this change to the possible drug immunosuppressive effect.

While antibiotics remain effective, their overuse promotes resistance and may alter host physiology (Trîncă et al. 2015). In contrast, *B. aegyptiaca* offers a holistic alternative, balancing antimicrobial action with host safety. Its ability to reduce pathogen burden without hematological disruption supports its application as a protective feed additive or therapeutic supplement.

Bashir et al. 2020 study investigating the effects of an aqueous stem bark extract mixture containing *B. aegyptiaca* on starter broiler chicks revealed promising in vivo antimicrobial activity. Supplementation of up to 80 ml/L in drinking water led to a marked reduction in *E. coli* counts and an increase in beneficial *Lactobacilli* populations in the caecum. Additionally, treated birds exhibited improved growth performance, enhanced immune responses, and elevated antioxidant enzyme activity, all without any adverse health effects, highlighting the extract’s potential as a safe and effective natural antimicrobial agent in poultry production.

## Conclusion

The present study provides compelling evidence supporting the therapeutic potential of *Balanites aegyptiaca* in poultry health management. The findings of this study highlight the broad therapeutic potential of *B. aegyptiaca* in animal health applications. Fruit extract and seed oil exhibited strong antioxidant capacity, which may contribute to mitigating oxidative stress during infections. The fruit extract also showed notable antiviral activity and selective cytotoxic effects against colorectal and pancreatic cancer cells, with minimal toxicity to normal cells. In poultry models, *B. aegyptiaca* extract significantly reduced mortality, clinical symptoms, and bacterial colonization in broilers experimentally infected with *E. coli* O78 and *C. perfringens* type A. Additionally, the extract maintained hematological stability, confirming its safety and potential as a natural alternative to synthetic antimicrobials and growth promoters in poultry health management.

### Future recommendation

Based on the present findings, *Balanites aegyptiaca* emerges as a promising natural candidate for inclusion in poultry health management programs. It’s demonstrated efficacy in reducing pathogenic load, while preserving normal hematological parameters, underscores its potential as a safe and effective feed additive or therapeutic supplement. Incorporating *B. aegyptiaca* into poultry diets could contribute to improved disease control and help mitigate the reliance on conventional antibiotics. However, further investigations are warranted to assess its safety profile, particularly at higher concentrations, as preliminary data suggest the potential for cytotoxic effects.

## Data Availability

All data obtained from the research work were included in the manuscript and owned by the authors, no permissions are required.
